# Orofacial Pain Is Associated With Oral Health–Related Quality of Life: The Tromsø Study 2015-2016

**DOI:** 10.1016/j.identj.2025.109328

**Published:** 2025-12-10

**Authors:** Amalie Storaa Jensen, Ólöf Anna Steingrímsdóttir, Birgitta Jönsson, Audun Stubhaug, Christopher Sivert Nielsen, Elin Hadler-Olsen

**Affiliations:** a The Public Dental Health Service of Troms County, Sjøvegan, Norway; b Department of Physical Health and Ageing, Norwegian Institute of Public Health, Oslo, Norway; c Department of Research, Oral Health Centre of Expertise in Eastern Norway, Oslo, Norway; d The Public Dental Health Service Competence Center of Northern Norway, Tromsø, Norway,; e Department of Periodontology, Institute of Odontology, The Sahlgrenska Academy, University of Gothenburg, Gothenburg, Sweden; f Department of Pain Management and Research, Oslo University Hospital, Oslo, Norway; g Institute of Clinical Medicine, University of Oslo, Oslo, Norway; h Department of Chronic Diseases, Norwegian Institute of Public Health, Oslo, Norway; i Department of Medical Biology, Faculty of Health Sciences, UiT The Arctic University of Norway, Tromsø, Norway

**Keywords:** Orofacial pain, Patient-reported outcomes, Oral health–related quality of life, Oral impact on daily performances, Population-based study

## Abstract

**Introduction and aims:**

Pain may have a negative impact on the quality of life. The purpose of this study was to evaluate the association between orofacial pain (OFP) and oral health–related quality of life (OHRQoL) among Norwegian adults.

**Methods:**

Data for this cross-sectional study were from the seventh Tromsø study (2015-2016). All residents 40 years and older in Tromsø municipality were invited. We had data on OHRQoL and OFP for 19,569 participants (40-96 years old, 52.2% women). Participants completed questionnaires on socioeconomic status and health. OHRQoL was evaluated by the Oral Impacts on Daily Performance (OIDP) questionnaire, whereas OFP was assessed by the Graphical Index of Pain. The association between OFP and OHRQoL was analysed by cross-tabulations and χ^2^ tests, as well as by multivariate binary logistic regression analysis to adjust for confounders.

**Results:**

Participants with OFP reported reduced OHRQoL compared to those without OFP. They experienced more problems with all activities assessed by the OIDP questionnaire than those without OFP, particularly with sleep/relaxation (24.6% vs 5.2%), performing daily tasks/work (11.5% vs 3.0%), and maintaining emotional stability (17.1% vs 5.1%). In multivariate regression, those with OFP had 3.44 times higher odds (95% CI, 2.77-4.27) of reporting reduced OHRQoL.

**Conclusion:**

The strong association between OFP and OHRQoL, particularly related to sleep, emotional stability, and performing daily tasks, highlights the interconnectedness of oral and general health. The requirement to attend a research station caused underrepresentation of groups with serious illnesses, which reduces the generalisability of the results. As the study design is cross-sectional, the associations observed may not be causal.

**Clinical relevance:**

This study shows the significant impact of OFP on OHRQoL. The findings highlight the importance of addressing OFP in clinical practice to improve the overall quality of life. These results emphasize the need for integrated approaches to oral and general health care.

## Introduction

Pain is a global health issue affecting a significant portion of the world’s population.^1^ A systematic review, including meta-analyses, found the prevalence of chronic pain to range from 9% to 64%, with a pooled mean of 31%.^2^ This variation in prevalence can partly be attributed to differing methodologies and definitions across studies, including methods for classifying pain.^2^ Pain may have severe consequences. At a societal level, individuals in pain take hold of extensive health care, financial, and social resources.^3^^,^^4^ On a personal level, pain can diminish quality of life (QoL) by increasing strain, disturbing sleep, and limiting physical and social activities.^5^ Those experiencing pain are also more likely to have mental disorders, such as depression and anxiety.^6^ In the orofacial region, masticatory muscle pain has been associated with anxiety, depression, and central pain sensitisation.^7^ Furthermore, pain can impair a person’s ability to work, leading to increased sick leave, risk of job loss, early retirement, or career changes.^5^

Pain is commonly evaluated using patient-reported outcome measures^8^ such as oral health–related quality of life (OHRQoL). OHRQoL assesses how oral health’s physical, psychological, and social dimensions impact QoL, serving as an indicator of overall health.^9^ OHRQoL is often evaluated through structured questionnaires such as the Oral Health Impact Profile–14 and the Oral Impacts on Daily Performances (OIDP).^10^^,^^11^ These tools are valuable as they provide insights to improve knowledge-based guidelines, health care system priorities, and public policy.^12^

Literature indicates that pain can negatively impact health-related QoL.^13^ Orofacial pain (OFP) conditions, such as temporomandibular dysfunction (TMD), have also been found to negatively impact OHRQoL.^14^ A systematic review with meta-analyses from 2020 found that 4 specific OFP conditions moderately impacted OHRQoL.^15^ The review included 39 original articles in the meta-analysis, with a total of 5849 patients. However, most of the studies involved fewer than 100 individuals and often selected help-seeking patient groups and not the general population. A more recent study also reported associations between OFP, poor sleep quality, and reduced satisfaction with life,^16^ but this also included a limited number of patients seeking help for OFP and no general population sample. This impairs the generalisability of the results as help-seeking patient groups may significantly differ from non-help-seekers,^17^ and the knowledge of the association between OFP and OHRQoL in the general population is limited. Therefore, this study aimed to further explore the relationship between OFP and OHRQoL in a large, general adult population in Norway. We assessed the associations between OFP and individual aspects of OHRQoL, as well as the overall association between OFP and OHRQoL, while controlling for sociodemographic and general health variables.

## Materials and Methods

### Study design and population

Data were from the seventh Tromsø Study (Tromsø7) conducted in 2015-2016.^18^ Invitations were sent to all residents aged 40 and above in Tromsø municipality, Norway, of whom 21,083 (65%) participated. Complete data on the outcome and exposure variables were available for 19,569 participants, forming the study cohort. The Tromsø Study is a broad, population-based health survey that is designed to assess numerous outcomes. Thus, no specific power analyses were done prior to the study, but the large number of participants ensures a power that is sufficient to assess outcomes, even with relatively small effect sizes. Participants underwent clinical examinations and completed questionnaires covering sociodemographic factors, lifestyle choices, and health-related issues, including detailed pain assessments. The study adhered to the Declaration of Helsinki, and all participants provided informed, written consent. Approval was granted by the Regional Committee for Health Research Ethics (REK320778).

### Outcome variable—the OIDP

OHRQoL was evaluated using the Norwegian version of the OIDP questionnaire, an 8-item instrument validated in a general Norwegian population.^16^ Participants reported how often during the past 6 months they had experienced difficulties, due to dental or oral problems, with 8 activities: eating and enjoying food, speaking and expressing themselves clearly, cleaning teeth, sleeping and relaxing, showing teeth without embarrassment, emotional stability, enjoying company with others, and performing daily tasks/work. Each activity had 5 response options: 0, never; 1, less than monthly; 2, once or twice a month; 3, once or twice a week; and 4, every or almost every day. For analysis, responses were dichotomised into no problems (option 0) and problems (options 1-4) for each item and cumulatively for all items, a common approach for this questionnaire.^19^, ^20^, ^21^, ^22^ Participants reporting problems with at least 1 activity were said to have a reduced OHRQoL.

### Exposure variable—orofacial pain

OFP was assessed with the Graphical Index of Pain tool.^23^ This is an online instrument featuring a hierarchical body map with 10 first-tier regions, including the head, and detailed second-tier regions. Participants were directed to identify any regions where they had experienced pain in the past 4 weeks, excluding transient pain and menstrual pain. When a first-tier region was marked, a detailed map of second-tier anatomic sites appeared, prompting participants to indicate all secondary sites where pain had been felt. Research assistants were available to help with the use of the Graphical Index of Pain tool if necessary. This study focused on pain in orofacial second-tier regions, including the temporomandibular joints; face below the eyes, nose, and lips; dentoalveolar regions; and oral mucosa, including the lips and throat. OFP was dichotomized into the following: 0 (no pain when none of the included second-tier regions were marked) and 1 (pain when 1 or more of the included second-tier regions were marked).

### Covariates

We included available covariates that, based on previous research, may confound the association between OFP and OHRQoL: sex,^24^ age,^25^ financial situation,^26^ self-reported general health,^27^ oral health,^28^ psychological distress,^29^^,^^30^ and number of dental visits.^31^ Data on age and sex were from the National Population Register. Age was categorised into 4 groups: (1) 40 to 49 years, (2) 50 to 59 years, (3) 60 to 69 years, and (4) 70 years or older in cross-tabulations and further dichotomised into (1) 40 to 59 years and (2) 60 years or older for regression analyses. Other covariates were from questionnaires.^32^ Participants were asked to rate their finances on a 5-point scale: 1, very difficult; 2, difficult; 3, average; 4, good; and 5, very good. Participants also assessed their general and oral health on a 5-point scale: 1, very poor; 2, poor; 3, neither poor nor good; 4, good; and 5, very good. Psychological distress was measured using the 10-item Hopkins Symptom Checklist, where participants indicated to what extent they had been bothered by 10 symptoms of anxiety and/or depression over the past 2 weeks on a scale from 1 = not bothered to 4 = very bothered. The average score was calculated and categorized into (1) no/light psychological distress (<1.85) and (2) psychological distress (≥1.85), which has been validated for the general Norwegian population.^33^ Participants also reported dental visits in the past 12 months, with responses categorized as (1) 0 visits, (2) 1 to 2 visits, and (3) ≥3 visits.

### Statistical analyses

Statistical analyses were conducted using the Statistical Package for Social Sciences (SPSS) for Windows, version 28 (IBM Corporation). The internal reliability of the OIDP questionnaire was measured by Cronbach’s α. Cross-tabulations examined group differences, and χ^2^ tests determined statistical significance. Further tests for variables with more than 2 categories were deemed unnecessary due to distinct differences between all variable categories. Multivariate binary logistic regression analyses explored the association between OHRQoL as the outcome and OFP as the exposure variable, with various sociodemographic and self-reported health measures as covariates. Tests for multicollinearity among all explanatory variables indicated no issues (tolerance <1, variance inflation factor <1.2, and no bivariate correlations >0.26 using Spearman’s ρ). We also examined multiplicative interactions between OFP and covariates on OHRQoL by incorporating cross-product interaction terms in the models. A regression model that included a cross-product term between OFP and sex and OFP and emotional distress was analysed, alongside models stratified by sex and emotional distress. Results are presented as odds ratios (ORs) and 95% confidence intervals (CIs) for experiencing problems with at least one of the daily activities included in the OIDP questionnaire. *P* values less than .05 were considered statistically significant. Data were missing for <2% of participants for all covariates except psychological distress (3.8% missing) and dental visits (6.6% missing). Missing values for covariates were omitted from the statistical analyses.

## Results

The age of the 19,569 participants in this study ranged from 40 to 96 years, with 52.2% being women. Table 1 shows the prevalence of reduced OHRQoL, defined as reporting problems with at least one of the activities assessed by the OIDP instrument. Overall, 21.2% of the participants had reduced OHRQoL. Most participants experienced issues with eating and enjoying food (14.1%), showing their teeth without being embarrassed (9.1%), and cleaning their teeth (7.9%). The rarest reported problems were with speaking (4.3%) and performing daily tasks/work (3.3%, Table 1).

**Table 1:** Oral health–related quality of life* (OHRQoL) for the complete cohort and stratified on orofacial pain.

	All, No. (%)	Orofacial pain,† No. (%)		
	(N = 19,569)	No (n = 18,779)	Yes (n = 790)	*P* value
OHRQoL total*				
No problem	15,413 (78.8)	15,015 (80.0)	398 (50.4)	<.001
Problem	4156 (21.2)	3764 (20.0)	392 (49.6)	
**Eat, enjoy food**				
No problem	16,793 (85.9)	16,291 (86.8)	502 (63.7)	<.001
Problem	2766 (14.1)	2480 (13.2)	286 (36.3)	
**Speak**				
No problem	18,696 (95.7)	17,969 (95.8)	727 (92.3)	<.001
Problem	850 (4.3)	789 (4.2)	61 (7.7)	
**Clean teeth**				
No problem	17,988 (92.1)	17,368 (92.6)	620 (79.0)	<.001
Problem	1548 (7.9)	1383 (7.4)	165 (21.0)	
**Sleep and relax**				
No problem	18,370 (94.1)	17,777 (94.8)	593 (75.4)	<.001
Problem	1162 (5.9)	969 (5.2)	193 (24.6)	
**Show teeth**				
No problem	17,765 (90.9)	17,112 (91.3)	653 (82.9)	<.001
Problem	1772 (9.1)	1637 (8.7)	135 (17.1)	
**Emotionally stabile**				
No problem	18,429 (94.4)	17,779 (94.9)	650 (82.9)	<.001
Problem	1090 (5.6)	956 (5.1)	134 (17.1)	
**Enjoy company with others**				
No problem	18,276 (93.6)	17,625 (94.1)	651 (82.8)	<.001
Problem	1243 (6.4)	1108 (5.9)	135 (17.2)	
**Daily tasks/work**				
No problem	18,862 (96.7)	18,166 (97.0)	696 (88.5)	<.001
Problem	647 (3.3)	557 (3.0)	90 (11.5)	

⁎ Measured by Oral Impacts on Daily Performances.

† Measured by the Graphical Index of Pain. The *P* value indicates statistically significant differences between participants with and without orofacial pain, calculated by the χ^2^ test.

The prevalence of reduced OHRQoL was 49.6% for participants with OFP and 20.0% for those without OFP (*P* < .001, Table 1). Eating-related problems were most common in both groups (36.0% of participants with OFP and 13.1% of those without OFP). The most pronounced differences between participants with and without OFP were in problems with sleeping and relaxing (24.6% vs 5.2%, respectively), performing daily tasks/work (11.5% vs 3.0%, respectively), and emotional stability (17.1% vs 5.1%, respectively). The smallest differences were in issues related to speaking and showing teeth without being embarrassed.

The percentage of participants who reported reduced OHRQoL (assessed with the OIDP questionnaire) in relation to the included covariates is presented in Table 2. The prevalence of reduced OHRQoL varied significantly across all covariates included in the analyses (*P* < .001, Table 2). Higher prevalence rates were observed among men, younger participants, those with an unfavourable financial situation, and participants reporting very poor or poor general or dental health, or symptoms of psychological distress. Participants who visited dental health services 1 to 2 times in the previous year had a lower prevalence of reduced OHRQoL compared to those who had no visits or more than 3 visits (16.3%, 22.2%, and 37.5%, respectively).

**Table 2:** Characteristics of participants and the proportion who reported reduced oral health–related quality of life (OHRQoL).

Characteristic	Total No. (N = 19,569)	Reduced OHRQoL,* No. (%) (n = 4156; 21.2%)	*P* value
**Sex**			
Women	10,212	2059 (20.2)	<.001
Men	9357	2097 (22.4)	
**Age group**			
40-49	6080	1444 (23.8)	<.001
50-59	5673	1213 (21.4)	
60-69	4786	954 (19.9)	
70+	3030	545 (18.0)	
**Finances**			
Very good	3428	507 (14.8)	
Good	10,324	1956 (18.9)	<.001
Average	5074	1349 (26.6)	
Difficult	575	264 (45.9)	
Very difficult	110	66 (60.0)	
**General health**			
Very good	2909	2510 (21.3)	
Good	10,534	2051 (19.5)	<.001
Moderate	4943	1299 (26.3)	
Poor	967	357 (36.9)	
Very poor	59	21 (35.6)	
**Psychological distress**			
No/mild	17,195	3457 (19.5)	<.001
Moderate/severe	1633	660 (38.8)	
**Dental health**			
Very good	2911	194 (6.7)	
Good	7733	982 (12.7)	<.001
Moderate	6750	1829 (27.1)	
Poor	1356	781 (57.6)	
Very poor	472	311 (65.8)	
**Dental visits**			
0	4488	995 (22.2)	<.001
1-2	10,728	1749 (16.3)	
≥3	3058	1148 (37.5)	
**Orofacial pain**†			
No	18,779	3764 (20.0)	<.001
Yes	790	392 (49.6)	

⁎ Reduced OHRQoL column shows the percentage of participants within each variable category reporting problems with at least one of the activities assessed by the Oral Impacts on Daily Performances instrument.

† Orofacial pain was assessed with the Graphical Index of Pain. *P* value shows the statistical significance of differences between variable categories calculated by the χ^2^ test.

Supplementary Table 1 shows patient characteristics by the prevalence of OFP. Patient characteristics varied significantly (*P* < .001) with prevalence of OFP in the same pattern as for prevalence of reduced OHRQoL (Supplementary Table 1).

### Regression analyses

Multivariate binary regression analyses were conducted to assess the association between OHRQoL (OIDP) and OFP while controlling for putative confounding factors. Significant interactions were found between OFP and sex (*P* = .022) and between OFP and emotional distress (*P* = .011). Consequently, 3 different regression models were run: (1) with all participants including interaction terms (OFP × sex and OFP × psychological distress), (2) stratified by sex, and (3) stratified by psychological distress (Table 3). The association between OFP and OHRQoL was strong and highly significant in all models (*P* < .001). Participants with OFP had 3.44 times higher odds of having reduced OHRQoL than participants without OFP (95% CI, 2.77-4.27). Sex-stratified analyses indicated that the association between OFP and OHRQoL was somewhat stronger among men (OR, 3.89; 95% CI, 2.79-5.43) than women (OR, 2.98; 95% CI, 2.44-3.63). When stratified by psychological distress, the association between OFP and OHRQoL was stronger among participants without psychological distress (OR, 3.61; 95% CI, 2.98-4.38) compared to those with distress (OR, 2.31; 95% CI, 1.62-3.29). Other covariates in the models were also significantly associated with OHRQoL, except for general health, which was not statistically significant in any of the models.

**Table 3:** Multivariate regressions for odds of reporting reduced oral health–related quality of life* (OHRQoL) for the complete cohort and stratified on sex and symptoms of psychological distress.

Characteristic	All, OR (95% CI)†	Women, OR (95% CI)‡	Men, OR (95% CI)‡	No psychological distress, OR (95% CI)§	Psychological distress, OR (95% CI)§
**Orofacial pain**¶					
No	Reference	Reference	Reference	Reference	Reference
Yes	**3.44 (2.77-4.27)**	**2.98 (2.44-3.63)**	**3.89 (2.79-5.43)**	**3.61 (2.98-4.38)**	**2.31 (1.62-3.29)**

OR, odds ratio.

Numbers in bold indicate *P* < .001.

⁎ Assessed by the Oral Impact on Daily Performances instrument.

† Adjusted for sex, age group, finances, general health, psychological distress, dental health, dental visits, orofacial pain × sex, and orofacial pain × psychological distress.

‡ Adjusted for age group, finances, general health, psychological distress, dental health, and dental visits.

§ Adjusted for sex, age group, finances, general health, dental health, and dental visits.

¶ Orofacial pain was assessed with the Graphical Index of Pain.

## Discussion

This study analysed the association between OFP and OHRQoL in a large, general adult population from northern Norway. After adjusting for sociodemographic and health-related variables, participants with OFP had 3.4 times higher odds of reporting problems with at least one of the activities assessed with the OIDP instrument than participants without OFP, demonstrating a strong association between OFP and OHRQoL.

We found that participants who reported OFP had a higher prevalence of problems with all the activities assessed by the OIDP instrument compared to those without OFP, with the largest differences observed in issues related to sleeping and relaxing, emotional stability, and performing daily tasks/work. Our findings are consistent with previous studies that report a strong association between pain and sleep disturbances.^34^ A meta-analysis based on studies using polysomnographic recording of sleep found a 44% prevalence of sleep disorders among patients with chronic pain.^35^ A study on patients with TMD found that 50% experienced poor sleep quality.^16^ Pain can cause cognitive arousal, impairing the ability to fall asleep^36^ and leading to disrupted sleep.^35^ However, the association between sleep and pain is bidirectional, and the temporal effect of sleep on pain may be as strong or stronger than the opposite.^34^ The mechanisms are complex, but a 2006 meta-analysis suggested that sleep disturbances may increase pain sensitivity and impair the effect of analgesic treatment.^37^ Both pain and insomnia are stressful and may challenge emotional stability. As for pain and sleep, there is a bidirectional association between mental health and sleep, where the impact of insomnia on mental health seems to be stronger than the reverse.^29^ Thus, pain, sleep, and emotional well-being are closely interconnected and may, at least partly, explain the association we found between OFP and the ability to perform daily tasks/work. Data from a qualitative study also support a close link between chronic pain, sleep disturbances, mood changes, and a negative effect on professional, family, and social life.^38^ As evidence points to sleep disturbances as a significant predisposing factor for both pain and mental illness, addressing insomnia in patients with OFP may alleviate pain and improve OHRQoL.

In the present study, we found that the odds of having reduced OHRQoL were higher among participants reporting symptoms of psychological distress than among those with no or few symptoms. Previous studies have found that symptoms of emotional distress, depression, and anxiety can strongly influence long-term outcomes of pain, including physical disability and work disability.^30^ Therefore, our finding that the association between OFP and OHRQoL was stronger among participants who did not experience emotional distress than those who did is surprising, and we can only speculate about the reasons. Psychiatric illness is a well-recognized risk factor for poor oral health, including caries and extensive tooth loss,^39^ with the latter known to have a significant impact on OHRQoL.^40^ Thus, participants with emotional distress may have additional oral health problems that affect their OHRQoL and may modulate the association between OFP and OHRQoL.

We found that a higher proportion of men than women experienced reduced OHRQoL, and the association between OFP and OHRQoL was somewhat stronger among men than women, as indicated by a significant interaction between sex and OFP on OHRQoL. Although studies have found that men have poorer oral health than women,^24^ the prevalence of pain in the orofacial region, most studied in the temporomandibular region, is often higher among women than men.^41^ Factors such as a lower experimental pain tolerance among women^42^ and that men may experience lower social acceptance to express and report pain than women^43^ could contribute to these sex differences. The latter may lead men to delay reporting pain until it has an intensity that more significantly affects their quality of life. In a previous study including this cohort, we also found that women sought dental care more often and more regularly than the men^44^ and, therefore, to a greater extent, may have gotten help handling the OFP.

The present study has some strengths and limitations. A key strength is that the Tromsø Study is a large, population-based study with a high attendance rate. However, a limitation is the underrepresentation of groups with serious illnesses or those living in nursing homes, due to the requirement to attend a research station, which reduces the generalisability of the study. The regression models used in this study, particularly those stratified by sex and psychological distress, may have low power due to the small number of participants reporting OFP, and the results should be interpreted with care. As the study design is cross-sectional, the associations observed may not be causal. Specific diagnoses, including TMD, neuropathic pain, or dental diseases, were not assessed or controlled for; thus, the OFP group was heterogeneous, and different diagnoses may differentially impact OHRQoL. Additionally, it is possible that other covariates not included in the statistical models could influence our results, such as medication use, bruxism, and trauma. Our study relied on self-reported measures, which are prone to bias such as social desirability bias, recollection bias, and acquiescence bias, which should be kept in mind when interpreting the results.

## CONCLUSION

The study demonstrates a strong association between OFP and OHRQoL, particularly related to the ability to sleep and relax, maintain emotional stability, and perform daily tasks/work. Men were more likely to have reduced OHRQoL than women, and OFP was more strongly associated with OHRQoL among men. Despite a lower prevalence of reduced OHRQoL among participants without symptoms of psychological distress, they had a stronger association between OFP and OHRQoL than those reporting symptoms of psychological distress. Our findings highlight the strong interconnectedness of oral, general somatic, and mental health, and they support the idea that oral health should be considered an integral part of general health and QoL. However, future studies are needed that explore changes over time and causal relationships. Also, studies that include more precise information on specific diagnoses, pain locations, or duration may give a deeper understanding of associations between OFP and OHRQoL.

## Ethics approval and consent to participate

This study was performed in line with the principles of the Declaration of Helsinki. Approval was granted by the Regional Committee for Health Research Ethics (REK320778). Written informed consent was obtained from all individual participants included in the study.

## Availability of data and materials

The data that support the findings of this study are available from The Tromsø study, but restrictions apply to the availability of these data, which were used under license for the current study and so are not publicly available. Data are, however, available from the authors upon reasonable request and with permission of the Tromsø Study: The Tromsø Study | UiT.

## Funding

The authors declare that no funds, grants, or other support were received during the preparation of this manuscript.

## Author contributions

All authors contributed to the study conception and design. Material preparation, data collection, and analysis were performed by Elin Hadler-Olsen, Christopher Sivert Nielsen, Ólöf Anna Steingrímsdóttir, and Audun Stubhaug. The first draft of the manuscript was written by Amalie Storaa Jensen, and all authors commented on previous versions of the manuscript. All authors read and approved the final manuscript.

## Conflict of interest

The authors declare that they have no known competing financial interests or personal relationships that could have appeared to influence the work reported in this paper.

## Appendix Supplementary materials

Image, application 1

Image, application 2

## Footnotes

Supplementary material associated with this article can be found in the online version at doi:10.1016/j.identj.2025.109328.
