# Supplementary material for: Orofacial Pain Is Associated With Oral Health–Related Quality of Life: The Tromsø Study 2015-2016
Source: Int Dent J. 2025 Dec 10;76(1):109328. doi: 10.1016/j.identj.2025.109328 (PMC12753215; doi:10.1016/j.identj.2025.109328)
Supplement: Supplementary file 2 [file mmc2.docx]

**Supplementary table 1. Participant characteristics by orofacial pain (OFP)**

|  | **Total**  19569  n | **OFP^1^**  790 (4.0)  n (%) | **No OFP^1^**  18779 (96.0)  n (%) | p |
| --- | --- | --- | --- | --- |
| **Sex** |  |  |  |  |
| Women | 10212 | 593 (%.8) | 9619 (94.2) | <0.001 |
| Men | 9357 | 197 (2.1) | 9160 (97.9) |  |
| **Age-group** |  |  |  |  |
| 40-49 | 6080 | 236 (6.0) | 5713 (94.0) | <0.001 |
| 50-59 | 5673 | 262 (4.6) | 5411 (95.4) |  |
| 60-69 | 4786 | 119 (2.5) | 4667 (97.4) |  |
| 70+ | 3030 | 42 (1.4) | 2988 (98.6) |  |
| **Finances** |  |  |  |  |
| Very good | 3428 | 101 (2.9) | 3327 (97.1) |  |
| Good | 10324 | 331 (3.2) | 9993 (96.8) | <0.001 |
| Average | 5074 | 270 (5.3) | 4804 (94.7) |  |
| Difficult | 575 | 70 (12.2) | 505 (87.8) |  |
| Very difficult | 110 | 17 (15.5) | 93 (84.5) |  |
| **General health** |  |  |  |  |
| Very good | 2909 | 43 (1.5) | 2866 (98.5) |  |
| Good | 10534 | 326 (3.1) | 10208 (96.9) | <0.001 |
| Moderate | 4943 | 265 (5.4) | 4678 (94.6) |  |
| Poor | 967 | 135 (14.0) | 832 (86.0) |  |
| Very poor | 59 | 11 (18.6) | 48 (81.4) |  |
| **Psychological distress** |  |  |  |  |
| No/mild | 17195 | 564 (3.3) | 16631 (96.7) | <0.001 |
| Moderate/severe | 1633 | 196 (12.0) | 1437 (88.0) |  |
| **Dental health** |  |  |  |  |
| Very good | 2911 | 92 (3.2) | 2819 (96.8) |  |
| Good | 7733 | 293 (3.8) | 7440 (96.2) | <0.001 |
| Moderate | 6750 | 261 (3.9) | 6489 (96.1) |  |
| Poor | 1356 | 96 (7.1) | 1260 (92.9) |  |
| Very poor | 472 | 44 (9.3) | 428 (90.7) |  |
| **Dental visits** |  |  |  |  |
| 0 | 4488 | 164 (3.7) | 4324 (96.3) | <0.001 |
| 1-2 | 10728 | 408 (3.8) | 10320 (96.2) |  |
| ≥3 | 3058 | 189 (6.2) | 2869 (93.8) |  |

^1^OFP = Orofacial pain assessed with the Graphical Index of Pain (GRIP). P-value shows statistical significance of differences between variable categories calculated by the Chi-square test.
